# Supplementary material for: Head magnetomyography (hMMG): A novel approach to monitor face and whole head muscular activity
Source: Psychophysiology. 2019 Nov 25;57(3):e13507. doi: 10.1111/psyp.13507 (PMC7027552; doi:10.1111/psyp.13507)
Supplement: Supplementary file 1 — Figure S1. (a) hMMG of the comparisons emotion vs. Neutral expression using MNE source reconstruction. (b) hMMG of the comparisons of emotion expressions against each other. Virtual sensors showing t‐values above |5| are displayed [file PSYP-57-e13507-s001.docx]

Supplementary Material

Supplementary Figure 1


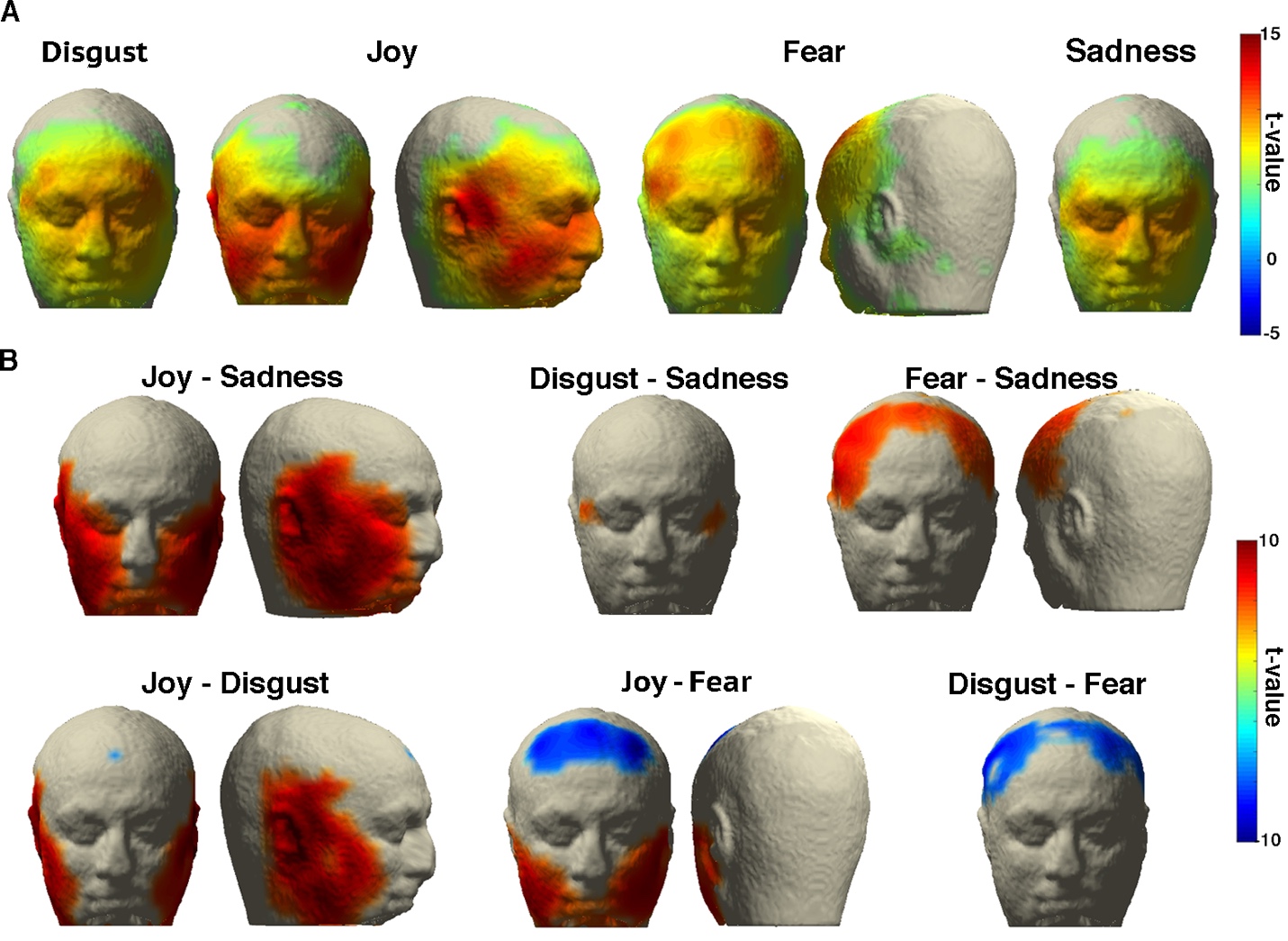


Supplementary Fig.1 . *(A)* **hMMG of the comparisons emotion vs. Neutral expression using MNE source reconstruction.** *(B)* **hMMG of the comparisons of  emotion expressions** against each other. Virtual sensors showing *t*-values above |5| are displayed.
